# Supplementary material for: Low Dielectric Properties and Transmission Loss of Polyimide/Organically Modified Hollow Silica Nanofiber Composites
Source: Polymers (Basel). 2022 Oct 21;14(20):4462. doi: 10.3390/polym14204462 (PMC9607524; doi:10.3390/polym14204462)
Supplement: Supplementary file 1 [file polymers-14-04462-s001.zip › polymers-1966297-supplementary.pdf]

Supplementary data

Table S1: Contact angle and water absorption of polyimide/m-HSNF composites.

Sample	Contact angle (degree)	Water absorption (%)
BPDA-BAPP	68.3	0.97
1 wt% BPDA-BAPP/m-HSNF	71.8	0.96
3 wt% BPDA-BAPP/m-HSNF	73.3	0.96
5 wt% BPDA-BAPP/m-HSNF	74.2	0.96
BPDA-HFBAPP	73.7	0.74
1 wt% BPDA-HFBAPP/m-HSNF	74.8	0.74
3 wt% BPDA-HFBAPP/m-HSNF	77.5	0.73
5 wt% BPDA-HFBAPP/m-HSNF	79.9	0.72
